# Diagnostic Test Criteria for HLA Genotyping to Prevent Drug Hypersensitivity Reactions: A Systematic Review of Actionable HLA Recommendations in CPIC and DPWG Guidelines

**DOI:** 10.3389/fphar.2020.567048

**Published:** 2020-09-23

**Authors:** Lisanne E. N. Manson, Jesse J. Swen, Henk-Jan Guchelaar

**Affiliations:** ^1^ Department of Clinical Pharmacy & Toxicology, Leiden University Medical Center, Leiden, Netherlands; ^2^ Leiden Network for Personalized Therapeutics, Leiden, Netherlands

**Keywords:** HLA genes, pharmacogenomics, hypersensitivity, antiepileptic drugs, abacavir, allopurinol, flucloxacillin

## Abstract

**Introduction:**

Certain HLA variants are associated with an increased risk of hypersensitivity reactions to specific drugs. Both the Clinical Pharmacogenetics Implementation Consortium (CPIC) and the Dutch Pharmacogenetics Working Group (DPWG) have issued actionable HLA gene – drug interaction guidelines but diagnostic test criteria remain largely unknown. We present an overview of the diagnostic test criteria of the actionable HLA – drug pairs.

**Methods:**

A systematic literature search was conducted in PubMed, Embase, Web of Science and Cochrane Library. Original case-control and cohort studies were selected and sensitivity, specificity, positive predictive value (PPV), negative predictive value (NPV) and number needed to genotype (NNG) were calculated for the actionable HLA-drug pairs.

**Results:**

In general, the HLA tests show high specificity and NPV for predicting hypersensitivity reactions. The sensitivity of HLA tests shows a wide range, from 0-33% for HLA-B*1502 testing to predict lamotrigine induced SJS/TEN up to 100% for HLA-B*5701 to predict immunologically confirmed abacavir hypersensitivity syndrome (ABC-HSR). PPV is low for all tests except for HLA-B*5701 and ABC-HSR which is approximately 50%. HLA-B*5701 to predict ABC-HSR shows the lowest NNG followed by HLA-B*5801 for allopurinol induced severe cutaneous adverse drug reactions and HLA-B*1502 for carbamazepine induced SJS/TEN.

**Discussion:**

This is the first overview of diagnostic test criteria for actionable HLA-drug pairs. Studies researching HLA genes and hypersensitivity are scarce for some of the HLA-drug pairs in some populations and patient numbers in studies are small. Therefore, more research is necessary to calculate the diagnostic test criteria more accurately.

## Introduction

Adverse drug reactions (ADRs) are an important cause of hospitalization and mortality in modern healthcare. ADRs can be classified as type A or type B. Type A reactions are often common and can be predicted from the drug’s pharmacological mechanism of action. Type B reactions, also known as idiosyncratic or hypersensitivity reactions, are usually much rarer and unpredictable and therefore pose a serious risk for patients as they can even be life-threatening.

The hypersensitivity reactions included in this review are: Stevens–Johnson syndrome (SJS) and toxic epidermal necrolysis (TEN), drug reaction with eosinophilia and systemic symptoms (DRESS), maculopapular exanthema (MPE), abacavir hypersensitivity syndrome (ABC-HSR) and drug-induced liver injury (DILI).

Stevens–Johnson syndrome (SJS) and toxic epidermal necrolysis (TEN) are the most serious types of severe cutaneous adverse drug reaction (SCAR). Both SJS and TEN are characterized by fever and mucocutaneous lesions leading to necrosis and sloughing of the epidermis. The two diseases are separated based on the percentage of body surface area detached: 1%–10% detachment defines SJS, 10%–30% detachment defines SJS/TEN overlap, and >30% detachment defines TEN. The mortality of SJS/TEN is estimated to be approximately 25%, ranging from 5%–10% for SJS to more than 30% for TEN (Sekula et al., 2013; Mahar et al., 2014; High, 2020; Mockenhaupt, 2020).

Drug reaction with eosinophilia and systemic symptoms (DRESS), also belonging to the term SCAR, describes a potentially life-threatening syndrome including a severe skin eruption, fever, hematologic abnormalities and involvement of internal organs. The mortality of DRESS is estimated to be about 5%–10% (Chen et al., 2010; Cacoub et al., 2011; Mockenhaupt, 2020). Another term used for DRESS is drug-induced hypersensitivity syndrome (DIHS).

Maculopapular exanthema (MPE) is a milder form of cutaneous ADR. It is the most common type of cutaneous ADR, occurring in approximately 2 percent of individuals exposed to drugs. MPE is characterized by erythematous macules and papules. Systemic symptoms include pruritus, low-grade fever, and mild eosinophilia. Usually the rash develops 5 to 14 days after starting treatment, but it may occur within one or two days. The rash usually improves within 2 weeks after withdrawal of the culprit drug.

Abacavir hypersensitivity (ABC-HSR) has several similar features as DRESS but does not have all the major criteria for DRESS. ABC-HSR is usually a combination of symptoms: Fever is almost always present and patients also often suffer from dizziness, headache, malaise and gastrointestinal symptoms. Respiratory symptoms and rash can be present as well. In the early use of abacavir, ABC-HSR became the main reason for drug discontinuation in approximately 8 percent of treated patients (Symonds et al., 2002; Young et al., 2008; Elizabeth J Phillips, 2020).

Drug-induced liver injury (DILI) is rare and has an estimated annual incidence between 10 and 15 per 10,000 to 100,000 persons exposed to prescription medications. Acute presentations of DILI include mild, asymptomatic liver test abnormalities but also liver failure. DILI accounts for approximately 10 percent of all cases of acute hepatitis. DILI is also a frequent reason for withdrawal of medications from the market (Larson, 2020).

The discovery of the first ADR-HLA genotype association, HLA-B*5701 associated with abacavir hypersensitivity, and its mandatory testing as obliged in the drug label initiated a whole new field of research leading to additional significant HLA-variant – ADR associations in the last decade.

Both the Clinical Pharmacogenetics Implementation Consortium (CPIC) and the Dutch Pharmacogenetics Working Group (DPWG) have issued actionable HLA gene – drug interaction guidelines. The HLA-drug pairs considered as actionable by CPIC and/or DPWG are: HLA-B*5701-abacavir, HLA-B*5701-flucloxacillin, HLA-B*5801-allopurinol, HLA-A*3101-carbamazepine, HLA-B*1511-carbamazepine, HLA-B*1502-carbamazepine, HLA-B*1502-oxcarbazepine, HLA-B*1502-lamotrigine and HLA-B*1502-phenytoin.

These guidelines are based upon different types of studies including case-control studies and cohort studies. While there is evidence for the association between HLA variants and the occurrence of ADRs, HLA testing is not yet being performed pre-emptively, except for HLA-B*5701 and abacavir and HLA-B*1502 and carbamazepine in some Asian populations. Pre-emptively testing HLA-A*3101 is recommended by the Canadian and Swiss drug label for some populations but it is not mandatory, We hypothesize that an important reason for a lack of implementation of pre-emptive HLA testing is the fact that diagnostic test criteria for the tests remain largely unknown. We have found one other review with an overview of diagnostic test criteria but this study has focused on antiepileptic drugs only and not on all drugs with actionable HLA gene – drug interactions (Mullan et al., 2019).

Therefore, we aim to present an overview of the diagnostic test criteria, namely the sensitivity, specificity, positive predictive value (PPV), negative predictive value (NPV) and the number needed to genotype (NNG) for all actionable HLA – drug pairs.

## Methods

We conducted a systematic literature search in PubMed, Embase, Web of Science and the Cochrane Library for case-control studies and prospective and retrospective cohort studies that evaluated known HLA – ADR associations. Based on the CPIC and DPWG guidelines for actionable HLA-drug pairs we restricted our search to articles concerning abacavir, allopurinol, flucloxacillin, carbamazepine, oxcarbazepine, lamotrigine and phenytoin. Search terms consisted of “*drug name” AND drug hypersensitivity AND HLA* and included synonyms of these terms (see **Supplementary File 1**). Records were screened on title and abstract. Duplicates, comments, editorials, narrative reviews, letters without original data and publications in languages other than English were excluded. Papers reporting original data with a minimum of 40 patients of which at least 10 hypersensitivity cases in total were included. We selected only studies where the HLA variants were genotyped directly. Studies that used a variant in linkage with the causal HLA variant were excluded since linkage disequilibrium is not known for all populations and shows considerable variation. We only selected studies where tolerant controls were available to calculate the diagnostic test criteria most accurately. All diagnostic test criteria were calculated by using the tolerant controls data while the population controls, if available, were used to calculate allele carrier frequencies in the general population. Calculation of sensitivity, specificity, PPV, NPV and NNG were done according to Tonk et al., and Steinberg et al. (Steinberg et al., 2009; Tonk et al., 2017) using the data described in the original articles. Considered endpoints included SCAR, SJS/TEN, DRESS, MPE, ABC-HSR and DILI. The endpoints used in the calculation of the diagnostic test criteria are similar to the endpoints mentioned in the specific CPIC and DPWG guidelines. For instance when the CPIC and/or DPWG guidelines only mention an association with SJS/TEN, other endpoints are not included in the results section.

To calculate the NPV, PPV and NNG, the incidence of the ADR is required. However, the incidence is not always available. Therefore we extracted the incidence from literature in one of the following ways and in this order:

- Directly from the included article itself when the article is a cohort study
- From the DPWG or CPIC guideline based on a review, meta-analysis or original article
- Derived from an original article in a similar population

In the tables in the result section, the source used for the incidence is mentioned.

## Results

### Study Selection

**Figure 1:**
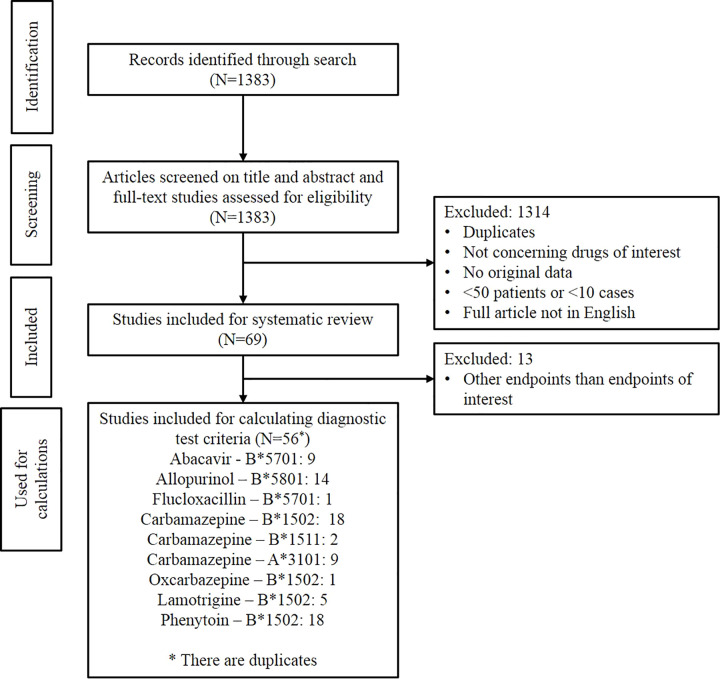
Study flow diagram of the systematic review inclusion.

**Figure 1** shows the result of the study selection. Initially, 1,383 publications were identified. The publications were first screened by title and abstract and then the full-text articles were assessed for eligibility. In total, 69 studies matched the inclusion criteria for analysis in this systematic review. Of these 69 studies, only the 56 studies investigating the same endpoints as the CPIC and/or DPWG guidelines and having the data needed for calculating the diagnostic test criteria were used in the *Results* section of this systematic review.

### Abacavir

Abacavir is a nucleoside analog reverse-transcriptase inhibitor (NRTI) used to treat and prevent HIV and is always used in combination with other antiretroviral drugs. To date, it is the only drug for which a pre-emptive HLA test is mandatory according to the drug label. Abacavir is contraindicated for patients who have been tested positive for the HLA-B*5701 variant or for patients who have not been tested because a positive test result is strongly associated with abacavir hypersensitivity syndrome (ABC-HSR) (Koninklijke Nederlandse Maatschappij ter bevordering der Pharmacie, 2020a). ABC-HSR occurs in 4%–8% of patients receiving abacavir prior to implementation of pre-emptive HLA-B*5701 testing (Hetherington et al., 2001; Mallal et al., 2008). The incidence of ABC-HSR seems to be much higher in Caucasian populations due to a higher frequency of the HLA-B*5701 allele than in non-Caucasian populations (To et al., 2013; Zhang H. et al., 2015; Youssoufi et al., 2017; Agbaji et al., 2019).

We have identified nine articles that met our inclusion criteria, all but one only in Caucasians. The results are summarized in **Table 1** for clinically diagnosed ABC-HSR and in **Table 2** for immunologically confirmed ABC-HSR.

**Table 1:** HLA-B*5701 and clinically diagnosed abacavir hypersensitivity reaction.

Article	Population	Description	Sensitivity	Specificity	PPV	NPV	Incidence	NNG	Frequency carriers in population (%)
Berka et al. (2012)	Canada	Prospective cohort study. N = 489	0.90	1	1	0.996	0.037 ^i^	27	4.1 (Berka et al., 2012)
Hetherington et al. (2002)	North America	Case-control study. 85 cases + 115 controls	0.554	0.988	0.666	0.980	0.043^iii^ (Hetherington et al., 2001)	42	7.2 (USA NMDP European Caucasian) (McCabe et al., 2020)
Hughes et al. (2004)	UK	Case-control study. 13 cases + 51 controls	0.462	0.902	0.153	0.978	0.037^iii^ (Symonds et al., 2002)	59	9.0 (England Blood Donors of Mixed Ethnicity) (McCabe et al., 2020)
Mallal et al. (2002)	West Australia	Cohort study. N = 200. 18 cases.	0.778	0.976	0.778	0.976	0.090 ^i^	14	8.7 (Mallal et al., 2002)
Martin et al. (2004)	West Australia	Cohort study. N = 248. Includes Mallal et al. (2002) patients. Mallal’s cases were reassessed using updated diagnostic criteria	0.947	0.983	0.818	0.996	0.073^i^	14	8.5 (Martin et al., 2004)
Mallal et al. (2008)	19 countries. Mostly white.	Randomized clinical trial. 980 prospectively genotyped + 976 control group retrospectively genotyped	0.455	0.976	0.612	0.955	0.078 ^i^	29	5.6 (Mallal et al., 2008)
Rauch et al. (2008)	Switzerland	prospective cohort study. N= 1,877 of which 149 suspected cases (of which 27 likely). 140 controls. Clinically suspected	0.309	0.986	0.244	0.990	0.0794^i^	41	6.0 (Rauch et al., 2008)
		Clinically likely	0.778	0.986	0.827	0.981	0.0144^i^	89	
Rodriguez et al. (2007)	Spain	case-control study. 26 cases + 27 controls.	0.423	0.963	0.491	0.952	0.078^iii^ (Mallal et al., 2008)	30	4.7 (Spain (Catalunya, Navarra, Extremadura, Aaragón, Cantabria),(McCabe et al., 2020)
Saag et al. (2008)	US White	case-control study. 130 white cases + 202 controls	0.442	0.960	0.492	0.952	0.080^iii^ (Brothers C et al., 2006)	28	7.2 (USA NMDP European Caucasian) (McCabe et al., 2020)
Saag et al. (2008)	US Black	case-control study. 69 black cases + 206 controls	0.145	0.990	0.358	0.969	0.036^iii^ (Brothers C et al., 2006)	192	1.4 (USA NMDP African American pop 2) (McCabe et al., 2020)

**Table 2:** HLA-B*5701 and immunologically confirmed abacavir hypersensitivity reaction.

Article	Population	Description	Sensitivity	Specificity	PPV	NPV	Incidence	NNG	Frequency carriers in population (%)
Mallal et al. (2008)	19 countries. Mostly white.	Randomized clinical trial. 980 prospectively genotyped. 976 control group (retrospectively genotyped)	1	0.969	0.479	1	0.027 ^i^	37	5.6 (Mallal et al., 2008)
Saag et al. (2008)	US White	Case control study. 130 white cases + 202 controls	1	0.960	0.406	1	0.026^i,iii^ (Brothers C et al., 2006)	38	7.2 (USA NMDP European Caucasian) (McCabe et al., 2020)
Saag et al. (2008)	US. Black	Case-control study. 69 black cases + 206 controls	1	0.990	0.206	1	0.0025 ^i,iii^ (Brothers C et al., 2006)	397	1.4 (USA NMDP African American pop 2) (McCabe et al., 2020)

Almost all the included studies were performed in Caucasians but the reported incidence of abacavir hypersensitivity differs between the studies. Therefore different incidences (1.4%–9.0% for Caucasian populations) are used for calculating the PPV, NPV, and NNG.

The specificity of the HLA-B*5701 test is high in all studies: 90-100%, as is the NPV: 95%–100% in clinically diagnosed and 100% in immunologically confirmed subjects. The sensitivity of the HLA-B*5701 test for abacavir HSR differs greatly between the studies and is between 31% and 90% for clinically diagnosed Caucasian patients. However, for immunologically confirmed HSR the sensitivity increases to 100%. The PPV is around 50%. The number of new abacavir users needed to genotype to prevent one case of ABC-HSR is 14–90 in Caucasians but ~10 times higher in Blacks.

### Allopurinol

Allopurinol is the most commonly used drug for the treatment of gout and hyperuricemia. However, a great safety concern of allopurinol is the risk of SCAR which is estimated to be 0.1%–0.4% among new users. (Hershfield et al., 2013) It has been proven that HLA-B*58:01 is associated with an increased risk for allopurinol induced SCAR. Nineteen studies are identified using the inclusion criteria described in the methods. Of these 19 studies, 14 are included in the results tables whereas the others investigated other endpoints or did not genotype cases and tolerant controls. The results of the HLA-B*5801 test criteria are shown in **Table 3** for SCAR, **Table 4** for DRESS, and **Table 5** for SJS-TEN.

**Table 3:** HLA-B*5801 and allopurinol induced severe cutaneous adverse drug reaction (SCAR).

Article	Population	Description	Sensitivity	Specificity	PPV	NPV	Incidence^ii,iii^	NNG	Frequency carriers in population (%)
Chen et al. (2015)	Eastern China	17 SCAR cases + 31 tolerant controls +120 population controls	0.882	0.935	0.0280	0.9997	0.0021	540	14.2 (Chen et al., 2015)
Cheng et al. (2015)	Han Chinese	92 SCAR cases + 75 tolerant controls + 99 population controls	0.946	0.880	0.0163	0.9999	0.0021	504	10.1 (Cheng et al., 2015)
Zhang X. et al. (2015)	Han Chinese	48 SCAR cases + 133 controls + 280 population controls	0.938	0.925	0.0256	0.9999	0.0021	508	12.1 (Zhang X. et al., 2015)
Chiu et al. (2012)	Hong Kong Han Chinese	19 SCAR cases + 30 controls	1	0.867	0.0155	1	0.0021	476	14.2 (Hong Kong Chinese) (McCabe et al., 2020)
Jung et al. 2011)	Korea	Retrospective cohort study. N = 448. 9 cases.	1	0.905	0.0217	1	0.0021	476	12.2 (Jung et al., 2011)
Kang et al. (2011)	Korea	26 SCAR cases + 57 controls	0.923	0.895	0.0181	0.9998	0.0021	516	11.8 (South Korea pop 10) (McCabe et al., 2020)
Gonçalo et al. (2013)	Portugal	25 SCAR cases + 23 controls	0.640	0.957	0.0300	0.9992	0.0021	744	4.0 (Portugal Center) (McCabe et al., 2020)
Cao et al. (2012)	Southern Han Chinese	16 SCAR cases + 63 controls	1	0.889	0.0186	1	0.0021	476	14.0 (Cao et al., 2012)
Chung et al. (2015)	Taiwan	48 cases + 138 controls	0.958	0.826	0.0115	0.9999	0.0021	497	20.0 (Taiwan Han Chinese) (McCabe et al., 2020)
Hung et al. (2005)	Taiwan	51 cases + 135 tolerant controls + 93 population controls	1	0.852	0.0140	1	0.0021	476	20.4 (Hung et al., 2005)
Ng et al. (2016)	Taiwan	106 cases + 285 controls	0.906	0.821	0.0105	0.9998	0.0021	526	20.0 (Taiwan Han Chinese) (McCabe et al., 2020)
Saksit et al. (2017)	Thailand	86 cases + 182 controls	0.965	0.885	0.0173	0.9999	0.0021	493	14.8 (Thailand) (McCabe et al., 2020)

**Table 4:** HLA-B*5801 and allopurinol induced drug reaction with eosinophilia and systemic symptoms (DRESS).

Article	Population	Description	Sensitivity	Specificity	PPV	NPV	Incidence^ii,iii^	NNG	Frequency carriers in population (%)
Kang et al. (2011)	Korea	21 DIHS cases + 57 controls	0.952	0.895	0.0045	1.0000	0.0005	2100	11.8 (South Korea pop 10) (McCabe et al., 2020)
Gonçalo et al. (2013)	Portugal	19 DRESS cases + 23 controls	0.632	0.957	0.0072	0.9998	0.0005	3167	4.0 (Portugal Center) (McCabe et al., 2020)
Cao et al. (2012)	Southern Han Chinese	3 DRESS cases + 63 tolerant controls + 572 population controls	1	0.889	0.0045	1	0.0005	2000	14.0 (Cao et al., 2012)
Chung et al. (2015)	Taiwan	22 DRESS cases + 138 controls	1	0.826	0.0029	1	0.0005	2000	20.0 (Taiwan Han Chinese) (McCabe et al., 2020)
Ng et al. (2016)	Taiwan	60 DRESS cases + 285 controls	0.917	0.821	0.0026	0.9999	0.0005	2182	20.0 (Taiwan Han Chinese) (McCabe et al., 2020)
Sukasem et al. (2016)	Thailand	6 DRESS cases + 100 tolerant controls +1095 population controls	1	0.960	0.0124	1	0.0005	2000	10.1 (Sukasem et al., 2016)
Saksit et al. (2017)	Thailand	19 DRESS cases + 182 controls	1	0.885	0.0043	1	0.0005	2000	14.8 (Thailand) (McCabe et al., 2020)

**Table 5:** HLA-B*5801 and allopurinol induced Stevens–Johnson syndrome (SJS)/toxic epidermal necrolysis (TEN).

Article	Population	Description	Sensitivity	Specificity	PPV	NPV	Incidence^i,iii^	NNG	Frequency carriers in population (%)
Kang et al. (2011)	Korea	5 SJS/TEN cases + 57 controls	0.800	0.895	0.0120	0.9996	0.0016	781	11.8 (South Korea pop 10) (McCabe et al., 2020)
Gonçalo et al. (2013)	Portugal	6 SJS/TEN cases + 23 controls	0.667	0.957	0.0240	0.9994	0.0016	938	4.0 (Portugal Center) (McCabe et al., 2020)
Cao et al. (2012)	Southern Han Chinese	13 SJS/TEN cases + 63 tolerant controls + 572 population controls	1	0.889	0.0142	1	0.0016	625	14.0 (Cao et al., 2012)
Chung et al. (2015)	Taiwan	26 SJS/TEN cases + 138 controls	0.923	0.826	0.0084	0.9999	0.0016	677	20.0 (Taiwan Han Chinese) (McCabe et al., 2020)
Ng et al. (2016)	Taiwan	46 SJS/TEN cases + 285 controls	0.891	0.821	0.0079	0.9998	0.0016	701	20.0 (Taiwan Han Chinese) (McCabe et al., 2020)
Sukasem et al. (2016)	Thailand	13 SJS-TEN cases + 100 tolerant controls + 1095 population controls	1	0.96	0.0385	1	0.0016	625	10.1 (Sukasem et al., 2016)
Tassaneeyakul et al. (2009)	Thailand	27 SJS/TEN cases + 54 controls	1	0.870	0.0122	1	0.0016	625	14.8 (Thailand) (McCabe et al., 2020)
Saksit et al. (2017)	Thailand	67 SJS/TEN cases + 182 controls	0.955	0.885	0.0131	0.9999	0.0016	654	14.8 (Thailand) (2020)

The incidence of SCAR is 0.1%–0.4% according to the CPIC guideline (Hershfield et al., 2013). Based on the results of a PubMed search the incidence of SJS/TEN is estimated to be 0.16% for SJS/TEN and 0.05% for DRESS (Sunicha Limkobpaiboon and Naruemon Dhana, 2010; Saokaew et al., 2014; Chong et al., 2018). Thus we use an incidence of 0.21% for SCAR (Min et al., 2015; Ke et al., 2019; Lin et al., 2019).

HLA-B*5801 testing in Asian populations shows high sensitivity and high specificity for allopurinol induced SCAR: 88%–100% and 82%–94% respectively. The only identified study in a non-Asian population, a Portuguese population, shows a lower sensitivity of 64% but a high specificity of 94%. The PPV is low (1.1% - 3.0%) while the NPV is approximately 1. The frequency of carriers of the HLA-B*5801 allele is lower in the Portuguese population than in Asian populations (4% versus 10%-20% respectively). If HLA-B*5801 is the only SNP associated with allopurinol induced SJS/TEN, the incidence of SCAR in this population is expected to be lower, leading to a higher NNG. In the Portuguese population also other HLA variants may be of importance. The calculated NNG for SCAR is 476-540 in Asian populations and is assumed to be much higher in the Portuguese population and other populations with a lower frequency of the HLA-B*5801 allele.

The high sensitivity and specificity are seen for both SJS/TEN and DRESS, as shown in **Table 4** (DRESS) and **Table 5** (SJS/TEN).

Sensitivity and specificity for the HLA-B*5801 test for allopurinol induced DRESS and SJS/TEN are comparable to the numbers mentioned above for SCAR. Sensitivity for allopurinol induced DRESS and SJS/TEN is 91.7%–100% and 80.0%–100% respectively in Asians. Specificity is between 82.1% and 96.0%. Due to the low incidence of allopurinol induced DRESS the NNG to prevent one case of DRESS is 2,000–2,182 in Asian populations. The NNG for allopurinol induced SJS/TEN is 625–781 in Asian populations.

### Flucloxacillin

Flucloxacillin is a penicillin antibiotic used for treating infections caused by Gram-positive bacteria such as staphylococci or streptococci. Although extremely rare, flucloxacillin has been associated with drug-induced liver injury (DILI). DILI is a collective term of different liver injuries as adverse drug reactions (ADRs) due to various drugs. The drug label of flucloxacillin mentions a clear correlation between HLA-B*5701 and flucloxacillin-induced liver damage but does not advise routine pre-emptive testing due to the rarity of DILI and the low PPV of 0.12% (Aurobindo Pharma, 2019). The incidence of DILI is estimated to be about 8.5/100,000 in new flucloxacillin users (Russmann et al., 2005). The DPWG has issued recommendations for HLA-B*5701 and flucloxacillin induced DILI. The recommendations indicate to monitor liver function more regularly and switch to an alternative when liver enzymes or bilirubin increase (Koninklijke Nederlandse Maatschappij ter bevordering der Pharmacie, 2020b). The CPIC however has not defined an actionable guideline about this drug-gene interaction.

Only one article is identified meeting our inclusion criteria. The results are shown in **Table 6**.

**Table 6:** HLA-B*5701 and flucloxacillin induced drug-induced liver injury (DILI).

Article	Population	Description	Sensitivity	Specificity	PPV	NPV	Incidence^iii^	NNG
Daly et al. (2009)	White European	51 DILI cases + 64 tolerant controls	0.843	0.938	0.0011	0.9999	0.000085	13953

The HLA-B*5701 test has relatively high sensitivity and specificity (84.3% and 93.8% respectively) for flucloxacillin induced DILI. However, due to the rarity of flucloxacillin induced DILI, the test has a very low PPV of 0.11% and high NPV (99.99%). The low incidence of DILI results in a high NNG for DILI of 13,953.

### Antiepileptic Drugs

Epilepsy is one of the most common chronic neurological disorders affecting millions of people worldwide. Many epileptic people use antiepileptic drugs (AEDs) to treat their condition. But these AEDs, especially carbamazepine, oxcarbazepine, phenytoin and lamotrigine are, along with allopurinol, the most common cause of cutaneous adverse drug reactions (cADRs) including the previously mentioned severe SJS/TEN and DRESS but also a milder form of cADR called macopapular exanthema (MPE). Carbamazepine, oxcarbazepine, lamotrigine and phenytoin induced cADRs are associated with HLA-B*1502 (Koninklijke Nederlandse Maatschappij ter bevordering der Pharmacie, 2020c; Koninklijke Nederlandse Maatschappij ter bevordering der Pharmacie, 2020d; Koninklijke Nederlandse Maatschappij ter bevordering der Pharmacie, 2020e; Koninklijke Nederlandse Maatschappij ter bevordering der Pharmacie, 2020f). For carbamazepine induced cADRs also associations with HLA-B*1511 and HLA-A*3101 have been shown.

There is evidence of cross-sensitivity among the AEDs carbamazepine, oxcarbazepine, lamotrigine and phenytoin (Bloch et al., 2014). In a Norwegian retrospective study of medical records it was found that phenytoin, carbamazepine and oxcarbazepine caused rashes in 27%–35% of patients with a history of another AED related rash (Alvestad et al., 2008). Lamotrigine was with 17% less involved in cross-sensitivity than carbamazepine, oxcarbazepine and phenytoin. A retrospective study of medical records in China also found high cross-sensitivity rates between the four AEDs, especially when carbamazepine and phenytoin were involved (Wang et al., 2010). There was a highly significant mutual risk for cross-sensitivity for CBZ and PHT, and OXC, and LTG. The substantial evidence for cross-sensitivity means caution is needed when prescribing these AEDs, especially when switching from one of these AEDs to another due to an cADR.

#### Carbamazepine

Carbamazepine is a widely used drug approved for the treatment of epilepsy, bipolar disorder and neuropathic pain. It is however known to be able to cause cADRs. Associations have been shown between HLA-B*1502 in Asian populations and SJS/TEN (Leckband et al., 2013; Koninklijke Nederlandse Maatschappij ter bevordering der Pharmacie, 2020c). Also in some populations HLA-B*1511 has been shown to be associated with SJS/TEN (Kim et al., 2011; Shi et al., 2012). In contrast, in European and Japanese populations not HLA-B alleles but HLA-A*3101 has been shown to be associated with SJS/TEN. This variant has also been shown to be associated with DRESS and MPE. The DPWG does not give an actionable advice on MPE, but the CPIC guideline on the other hand does state that an HLA-A*3101 positive carbamazepine user has a higher chance of MPE as well as SJS/TEN and DRESS. In the drug label HLA-B*1502 and HLA-A*3101 testing is advised in high risk patients. Patients are deemed to be at risk when they come from countries with a high prevalence of the HLA-B*1502 allele such as Hong Kong, Thailand, Taiwan, Malaysia, and parts of the Philippines. The drug label states the use of carbamazepine and other AEDs associated with SJS/TEN should be avoided in patients who test positive for the HLA-A*3101 or HLA-B*1502 alleles (ULC MP, 2015). The CPIC’s advice is the same whereas the DPWG guideline also advises to consider an alternative in HLA-B*1511 positive patients (Hershfield et al., 2013; Koninklijke Nederlandse Maatschappij ter bevordering der Pharmacie, 2020c). The Swiss and Canadian drug label strongly recommend testing HLA-A*3101 in high risk populations. The Swiss label considers Japanese, Caucasians, American indigenous population and patients of Spanish, Portuguese, South Indian and Arabic ancestry to be at high risk.

The incidence of SJS/TEN is 0.005% in European Caucasian populations (Genin et al., 2014; Koninklijke Nederlandse Maatschappij ter bevordering der Pharmacie, 2020c) and in Chinese and other some Asian populations it is 0.25% (Genin et al., 2014; Koninklijke Nederlandse Maatschappij ter bevordering der Pharmacie, 2020c). To be able to calculate NPV, PPV and NNG for other Asian populations such as Vietnamese and Malaysian, an incidence of 0.25% is assumed. The incidence of SJS/TEN in non-European Caucasians is assumed to be 0.005%.

The results of studies studying the association of HLA-B*1502 and carbamazepine induced SJS/TEN are summarized in **Table 7**.

**Table 7:** HLA-B*1502 and carbamazepine induced Stevens–Johnson syndrome (SJS)/toxic epidermal necrolysis (TEN).

Article	Population	Description	Sensitivity	Specificity	PPV	NPV	Incidence	NNG	Frequency carriers in population (%)
Amstutz et al. (2013)	Canada	42 cases (9 SJS/TEN, 6 HSS, 26 MPE, 1 AGEP) + 92 controls	0.333	0.989	0.0014	0.99996	0.00005	60000	No data available of Canadians or North Americans of mixed ethnicity. (McCabe et al., 2020)
Wu et al. (2010)	Central China	36 cases (8 SJS/TEN + 28 MPE) + 50 tolerant controls + 71 population controls	1	0.92	0.0303	1	0.0025	400	8.5 (Wu et al., 2010)
He et al. (2013)	China Northeast Han Chinese	35 SJS/TEN cases + 125 controls	0.229	0.984	0.0346	0.9980	0.0025	1750	3.8 (China North Han) (McCabe et al., 2020)
Shi et al. (2012)	China Southern Han Chinese	18 SJS/TEN cases + 93 controls	0.722	0.886	0.0160	0.992	0.0025	554	13.7 (China South Han) (McCabe et al., 2020)
Shi et al. (2017)	China Southern Han Chinese	56 SJS/TEN cases + 180 controls	0.696	0.844	0.0110	0.9991	0.0025	574	13.7 (China South Han) (McCabe et al., 2020)
Cheung et al. (2013)	Hong Kong Han Chinese	26 SJS/TEN cases + 135 controls	0.923	0.881	0.0191	0.9998	0.0025	433	17.9 (Hong Kong Chinese BMDR) (McCabe et al., 2020)
Khosama et al. (2017)	Indonesia	14 SJS/TEN cases + 53 controls	0.571	0.736	0.0054	0.9985	0.0025	700	22.9 (Indonesia Java Western) (McCabe et al., 2020)
Kim et al. (2011)	Korea	24 SCAR cases (7 SJS, 17 HSS) + 50 tolerant controls + 485 population controls	0.143	1	1	0.9979	0.0025	2800	0.4 (Kim et al., 2011)
Khor et al. (2017)	Malaysia	28 SJS/TEN cases + 227 controls	0.714	0.899	0.0174	0.9992	0.0025	560	
	Malaysia Chinese	6 cases + 106 controls	0.667	0.877	0.0134	0.9990	0.0025	600	11.3 (Malaysia Peninsular Chinese) (McCabe et al., 2020)
	Malaysia Indian	6 cases + 57 controls	0.333	0.965	0.0233	0.9983	0.0025	1200	5.2. (Malaysia Peninsular Indian) (McCabe et al., 2020)
	Malaysia Malaysian	16 cases + 64 controls	0.875	0.875	0.0172	0.9996	0.0025	457	22.3 (Malaysia Peninsular Malay) (McCabe et al., 2020)
Wang et al. (2011)	Southern Han Chinese	48 cases (9 SJS/TEN, 39 MPE) + 80 tolerant controls + 62 population controls	1	0.863	0.0179	1	0.0025	400	17.7 (Wang et al., 2011)
Chung et al. (2004)	Taiwan Han Chinese	44 SJS cases + 101 tolerant controls + 93 population controls	1	0.970	0.0781	1	0.0025	400	8.6 (Chung et al., 2004)
Hsiao et al. (2014)	Taiwan Han Chinese	194 cases (51 MPE, 112 SJS/TEN, 8 other) + 152 controls	0.884	0.928	0.0297	0.9997	0.0025	453	8.8 (Taiwan Han Chinese) (McCabe et al., 2020)
Kulkantrakorn et al. (2012)	Thailand	34 SJS/TEN cases + 40 controls	0.941	0.825	0.0133	0.9998	0.0025	425	16.1 (Thailand Northeast pop 2) (McCabe et al., 2020)
Locharernkul et al. (2008)	Thailand	15 cases (6 SJS, 9 MPE) + 42 controls	1	0.810	0.0130	1	0.0025	400	16.1 (Thailand Northeast pop 2) (McCabe et al., 2020)
Sukasem et al. (2018)	Thailand	38 cases (17 MPE, 16 SJS/TEN, 5 DRESS) + 271 tolerant controls + 470 population controls	0.75	0.959	0.0443	0.993	0.0025	533	15.1 (Sukasem et al., 2018)
Tassaneeyakul et al. (2010)	Thailand	42 SJS/TEN cases and 42 controls	0.881	0.881	0.0182	0.9997	0.0025	454	16.1 (Thailand Northeast pop 2) (McCabe et al., 2020)
Nguyen et al. (2015)	Vietnam	38 cases (20 SJS, 7 TEN, 8 SJS-TEN, 3 DRESS) + 25 controls	0.914	0.760	0.0095	0.997	0.0025	438	25,2 (Vietnam Hanoi Kinh pop 2) (McCabe et al., 2020)
Genin et al. (2014)	European	20 SJS/TEN cases, 10 DRESS cases + 43 tolerant controls from other study + 8862 population controls	0	1	–	1	0.00005	–	0.05 (Genin et al., 2014)
Genin et al. (2014)	Chinese from Taiwan	53 SJS/TEN cases, 10 DRESS cases + 72 tolerant controls + 710 population controls	0.774	0.44	0.033	0.994	0.0025	517	8.5 (Genin et al., 2014)

In general, studies find a relatively high sensitivity (67-100%) and specificity (73-100%) for HLA-B*1502 and carbamazepine induced SJS/TEN. However in some populations such as Korean, Canadians of diverse ancestry, Indonesian, North east Chinese and Indians in Malaysia lower sensitivities of 14-57% are found (Kim et al., 2011; Amstutz et al., 2013; He et al., 2013; Khor et al., 2017; Khosama et al., 2017). In these populations, the allele frequency is lower than in the other Asian populations which may indicate that in these populations also other HLA variants may be of importance, for instance the HLA-A*3101 or HLA-B*1511 variants. In the study from Genin et al. the sensitivity in Europeans is zero and the specificity is 100% since there were no Europeans positive for HLA-B*1502 in this study.

Due to the low incidence, the PPV is low (0.14%–7.8%) while the NPV is high (99.2%–100%). The NNG is 400–2,800 in Asian populations of which most studies lead to a NNG of 400–700. In Canadians, the NNG is 60,000.

Studies about the association of carbamazepine induced SJS/TEN and HLA-B*1511 are scarce. Only two studies meet our inclusion criteria. See **Table 8**.

**Table 8:** HLA-B*1511 and carbamazepine induced Stevens–Johnson syndrome (SJS)/toxic epidermal necrolysis (TEN).

Article	Population	Description	Sensitivity	Specificity	PPV	NPV	Incidence	NNG	Frequency carriers in population (%)
Kim et al. (2011)	Korea	24 SCAR cases (7 SJS, 17 HSS) + 50 tolerant controls + 485 population controls	0.429	0.96	0.0262	0.9985	0.0025	933	3.9 (Kim et al., 2011)
Shi et al. (2012)	China Southern Han Chinese	56 SJS/TEN cases + 180 controls	0.071	0.843	0.0011	0.9972	0.0025	5600	0.4 (China South Han) (McCabe et al., 2020)

In southern Han Chinese, the HLA-B*1511 test shows very low sensitivity (7.1%) for carbamazepine induced SJS/TEN. In a Korean population the sensitivity is higher but still low (42.9%). The low sensitivity in southern Han Chinese can be explained by the low allele frequency of HLA-B*1511 in this population (0.4%). The low sensitivity indicates that testing only HLA-B*1511 in carbamazepine initiators might be insufficient to predict SJS/TEN, because of the influence of other HLA-genes such as HLA-B*1502. Specificity in both studies is high and so is the NPV. The PPV is low. The NNG in the study by Kim et al. is 933 while in the study among Southern Han Chinese from Shi et al. it is 5,600 (Kim et al., 2011; Shi et al., 2012).

The results of carbamazepine induced SJS/TEN and HLA-A*3101 are summarized in **Table 9**.

**Table 9:** HLA-A*3101 and carbamazine induced Stevens–Johnson syndrome (SJS)/toxic epidermal necrolysis (TEN).

Article	Population	Description	Sensitivity	Specificity	PPV	NPV	Incidence	NNG	Frequency carriers in population (%)
Amstutz et al. (2013)	Canada	42 cases (9 SJS/TEN, 6 HSS, 26 MPE, 1 AGEP) + 92 controls	0	0.967	0	0.9999	0.00005	–	No data available of Canadians or North Americans of mixed ethnicity.
Hsiao et al. (2014)	Han Chinese from Taiwan	194 cases (51 MPE, 112 SJS/TEN, 8 other) + 152 controls (investigates associations)	0.018	0.967	0.0014	0.9974	0.0025	22400	5.5 (Taiwan Han Chinese) (McCabe et al., 2020)
Ozeki et al. (2011)	Japan	77 cADR cases (36 DIHS, 6 SJS/TEN, 35 other) + 420 controls	0.833	0.871	0.0160	0.9995	0.0025	480	16.1 (Japan pop 16) (McCabe et al., 2020)
Kim et al. (2011)	Korea	24 SCAR cases (7 SJS, 17 HSS) + 50 tolerant controls + 485 population controls	0.429	0.86	0.0076	0.9983	0.0025	933	10.3 (Kim et al., 2011)
Khor et al. (2017)	Malaysia	28 SJS/TEN cases + 227 controls	0.107	0.947	0.0051	0.9976	0.0025	3733	
	Malaysia Chinese	6 cases + 106 controls	0	0.972	0	0.9974	0.0025	–	2.6 (Malaysia Peninsular Chinese) (McCabe et al., 2020)
	Malaysia Indian	6 cases + 57 controls	0.500	0.912	0.0141	0.9986	0.0025	800	4.1 (Malaysia Peninsular Indian) (McCabe et al., 2020)
	Malaysia Malaysian	16 cases + 64 controls	0	0.938	0	0.9973	0.0025	–	0.8 (Malaysia Peninsular Malay) (McCabe et al., 2020)
Ihtisham et al. (2019)	North India	35 cases (27 MPE/6 SJS-TEN/2 DRESS) +70 controls	0	0.957	0	0.9974	0.0025	–	3.8 (India North pop 2) (McCabe et al., 2020)
Genin et al. (2014)	European	20 SJS/TEN cases, 10 DRESS cases + 257 tolerant controls + 8862 population controls	0.150	0.961	0.0002	0.99996	0.00005	133333	4.5 (Genin et al., 2014)
Genin et al. (2014)	Chinese from Taiwan	53 SJS/TEN cases, 10 DRESS cases + 72 tolerant controls + 710 population controls	0.019	0.958	0.0011	0,9974	0.0025	21200	3.7 (Genin et al., 2014)

HLA-A*3101 testing shows high specificity (86.0%–96.7%) but mostly low sensitivity (0.0%–83.3%) for carbamazepine induced SJS/TEN. Only in Japanese, Korean and Indian Malaysian relatively high sensitivities are found (42.9%–83.3%). The PPV is 0%–1.6% while the NPV is close to 100.0% in all studies. The NNG shows a high variability and is between 480 and 133,333.

The results of carbamazepine induced DRESS and HLA-A*3101 are summarized in **Table 10**. The incidence of DRESS is assumed to be 0.05% in both Asian and Caucasian populations (Koninklijke Nederlandse Maatschappij ter bevordering der Pharmacie, 2020c).

**Table 10:** HLA-B*3101 and carbamazepine induced drug reaction with eosinophilia and systemic symptoms (DRESS).

Article	Population	Description	Sensitivity	Specificity	PPV	NPV	Incidence	NNG	Frequency carriers in population (%)
Amstutz et al. (2013)	Canada	42 cases (9 SJS/TEN, 6 HSS, 26 MPE, 1 AGEP) + 92 controls	0.5	0.967	0.0075	0.9997	0.0005	4000	No data available of Canadians or North Americans of mixed ethnicity.
Genin et al. (2014)	Chinese from Taiwan	53 SJS/TEN cases, 10 DRESS cases + 72 tolerant controls + 710 population controls	0.5	0.958	0.0060	0.9997	0.0005	4000	3.7 (Genin et al., 2014)
Genin et al. (2014)	European	20 SJS/TEN cases, 10 DRESS cases + 257 tolerant controls from other study + 8,862 population controls	0.7	0.961	0,0089	0.9998	0.0005	2857	4.5 (Genin et al., 2014)
Hsiao et al. (2014)	Han Chinese from Taiwan	194 cases (51 MPE, 112 SJS/TEN, 8 other) + 152 controls	0.304	0.967	0.0046	0.9996	0.0005	6571	5.5 (Taiwan Han Chinese) (McCabe et al., 2020)
Ozeki et al. (2011)	Japan	77 cADR cases (36 DIHS, 6 SJS/TEN, 35 other) + 420 controls	0.583	0.871	0.0023	0.9998	0.0005	3429	16.1 (Japan pop 16) (McCabe et al., 2020)
Kim et al. (2011)	Korea	24 SCAR cases (7 SJS, 17 HSS) + 50 tolerant controls + 485 population controls	0.588	0.86	0.0021	0.0098	0.0005	3400	10.3 (Kim et al., 2011)
Itisham et al. (2019)	North India	35 cases (27 MPE/6 SJS-TEN/2 DRESS) +70 controls	0	0.957	0	0.9995	0.0005	–	3.8 (India North pop 2) (McCabe et al., 2020)

Sensitivity for HLA-A*3101 and carbamazepine induced DRESS is about 50% in several populations (30.4%–70.0%) while specificity is high (86%–97%). The exception is the study by Ihtisham et al. with a sensitivity of 0. However, this study only had two DRESS cases which both did not carry the HLA-A*3101 allele Ihtisham et al. (2019). The NNG is high in all studies (2,857–6,571).

The results of HLA-A*3101 and carbamazepine induced MPE are summarized in **Table 11**. The incidence of MPE is assumed to be 10% in Caucasian users and 4.4% in Chinese and other Asian users (Koninklijke Nederlandse Maatschappij ter bevordering der Pharmacie, 2020c).

**Table 11:** HLA-B*3101 and carbamazepine induced maculopapular exanthema (MPE).

Article	Population	Description	Sensitivity	Specificity	PPV	NPV	Incidence	NNG	Frequency carriers in population (%)
Amstutz et al. (2013)	Canada with diverse ethnic background	42 cases (9 SJS/TEN, 6 HSS, 26 MPE, 1 AGEP) + 92 controls	0.231	0.967	0.438	0.919	0.1	43	No data available of Canadians or North Americans of mixed ethnicity.
Li et al. (2013)	China Han Chinese	40 MPE cases + 52 controls + 72 population controls (allele frequency instead of carrier frequency)	0.013	0.990	0.056	0.956	0.044	1818	3.0 (China Sichuan HIV negative (McCabe et al., 2020)
McCormack et al. (2018)	European descended	95 MPE cases + 869 controls	0.168	0.969	0.376	0.913	0.1	59	4.6 (Poland BMR) (McCabe et al., 2020)
McCormack et al. (2018)	Han Chinese descended	85 MPE cases + 197 controls	0.043	0.940	0.032	0.955	0.044	523	12.2 (China Han HIV negative) (McCabe et al., 2020)
Hsiao et al. (2014)	Han Chinese from Taiwan	194 cases (51 MPE, 112 SJS/TEN, 8 other) + 152 controls (investigates associations)	0.137	0.967	0.161	0.961	0.044	166	5.5 (Taiwan Han Chinese) (McCabe et al., 2020)
Itisham et al. (2019)	North India	35 cases (27 MPE/6 SJS-TEN/2 DRESS) +70 controls	0.222	0.957	0.193	0.964	0.044	102	3.8 (India North pop 2) (McCabe et al., 2020)

Sensitivity for HLA-A*3101 and carbamazepine induced MPE is low (1%–23%). The sensitivity is higher in Europeans, Canadians of mixed ethnicities and Indian populations than in Chinese populations. Specificity is high in all populations (94%–99%). PPV is low (3%–44%) while NPV is consistently high (91%–98%). Due to the high incidence of MPE, the NNG is low, especially in Canadians and Europeans (Locharernkul et al., 2008; GlaxoSmithKline, 2009; US Food and Drug Administration, 2009; He et al., 2012; Shi et al., 2012; Amstutz et al., 2013; He et al., 2013; Lv et al., 2013; Caudle et al., 2014; Genin et al., 2014; ULC MP, 2015; Moon et al., 2016; Pfizer, 2016; Chen et al., 2017; Khor et al., 2017; Khosama et al., 2017; Deng et al., 2018; Fowler et al., 2019). But NNG is also low in North India (An et al., 2010) due to a relatively high sensitivity (22.2%). In the other study populations NNG is 166-1818.

#### Oxcarbazepine

Oxcarbazepine is an AED structurally related to carbamazepine. Therefore it might not be surprising that there is also evidence of an association of HLA-B*1502 and oxcarbazepine induced SJS/TEN leading to CPIC and DPWG guidelines about this association (Leckband et al., 2013; Koninklijke Nederlandse Maatschappij ter bevordering der Pharmacie, 2020e). The drug label of oxcarbazepine warns to only use the drug in HLA-B*1502 positive patients when the benefits clearly outweigh the risk (US Food and Drug Administration, 2009).

However, only four studies for oxcarbazepine and hypersensitivity are identified. Three of these four studies were studying oxcarbazepine induced MPE and only one study investigated oxcarbazepine induced SJS/TEN (He et al., 2012; Lv et al., 2013; Moon et al., 2016; Chen et al., 2017). This may be due to the fact oxcarbazepine-induced SJS/TEN is less common than carbamazepine-induced SJS/TEN. Results of the study can be found in **Table 12**. The incidence used for calculations is 0.0826% (Chen et al., 2017; Koninklijke Nederlandse Maatschappij ter bevordering der Pharmacie, 2020e).

**Table 12:** HLA-B*1502 and oxcarbazepine induced Stevens–Johnson syndrome (SJS)/toxic epidermal necrolysis (TEN).

Article	Population	Description	Sensitivity	Specificity	PPV	NPV	Incidence^i,iii^	NNG	Frequency carriers in population (%)
Chen et al. (2017)	Taiwan Han Chinese	50 cADR cases (20 SJS-TEN, 6 DRESS, 22 MPE, 2 BDFE) + 101 controls	0.706	0.921	0.0073	0.9997	0.000826	1715	8.8 (Taiwan Han Chinese) (McCabe et al., 2020)

Chen et al. investigated the association of HLA-B*1502 and oxcarbazepine induced cutaneous adverse drug reactions including SJS/TEN in Taiwanese and Thai Han Chinese. However, because there were only three Thai cases and no tolerant controls, we do not report this data. Sensitivity for Taiwanese Han Chinese is 70.6% and specificity is 92.1%. The NNG for Taiwanese Han Chinese 1715.

#### Lamotrigine

Lamotrigine is approved for epilepsy and for bipolar disorders. There is evidence HLA-B*1502 is associated with lamotrigine induced SJS/TEN. The drug label of lamotrigine does give a warning about serious skin rashes including SJS usually occurring within 2–8 weeks of initiation of lamotrigine. The incidence is higher in pediatric patients than adults. Interestingly, HLA-B*1502 is not mentioned in the drug label at all (GlaxoSmithKline, 2009).

We have identified nine articles about lamotrigine and hypersensitivity that meet our inclusion criteria. Of these only five articles report information about the association of HLA-B*1502 and lamotrigine induced SJS/TEN, the other studies investigate other endpoints such as DRESS or MPE. The results of the studies investigating SJS/TEN can be found in **Table 13**.

**Table 13:** HLA-B*1502 and lamotrigine induced Stevens–Johnson syndrome (SJS)/toxic epidermal necrolysis (TEN).

Article	Population	Description	Sensitivity	Specificity	PPV	NPV	Incidence^i^	NNG	Frequency carriers in population (%)
Kazeem et al. (2009)	European	22 cases (10 SJS, 12 HSR) + 43 controls	0	1	–	0.9990	0.001	–	0.0 (Bulgaria, Croatia, Czech Republic, Germany, Ireland Northern, Netherlands, Poland DKMS) (McCabe et al., 2020)
An et al. (2010)	Han Chinese	25 cases (3 SJS/TEN, 22 MPE) + 21 tolerant controls + 71 population controls	0.333	0.952	0.0070	0.9993	0.001	3000	8.5 (Koomdee et al., 2017)
Shi et al. (2011)	Han Chinese	14 cADR cases (2 SJS/TEN + 12 MPE) + 28 tolerant controls + 264 population controls	0	0.931	0	0.9989	0.001	–	14.1 (Shi et al., 2011)
Shi et al. (2017)	Southern Han Chinese	22 SJS cases + 102 controls	0.227	0.814	0.0012	0.9991	0.001	4400	13.7 (China South Han) (McCabe et al., 2020)
Koomdee et al. (2017)	Thailand	15 cADR cases (4 SJS, 1 dress, 10 MPE) + 50 tolerant controls + 986 population controls	0.25	0.88	0.0021	0.9991	0.001	4000	15.5 (Koomdee et al., 2017)

The incidence of lamotrigine induced SJS/TEN is assumed to be 0.1% (Koninklijke Nederlandse Maatschappij ter bevordering der Pharmacie, 2020d).

The HLA-B*1502 test shows low sensitivity (0%–33.3%) but high specificity (81.4%–100%) in various populations. the NNG is with 3,000–4,400 much higher than for carbamazepine and oxcarbazepine.

#### Phenytoin

Phenytoin is an AED but is in some countries also approved as a class 1b antiarrhythmic. There is evidence HLA-B*1502 is associated with phenytoin induced SJS/TEN as mentioned in the DPWG and CPIC guidelines (Caudle et al., 2014; Koninklijke Nederlandse Maatschappij ter bevordering der Pharmacie, 2020f). Also the drug label mentions this association but states that the evidence is weak. The label mentions consideration should be given to avoid phenytoin as an alternative for carbamazepine in patients positive for HLA-B*1502 (Pfizer, 2016).

We have identified 10 articles about phenytoin and hypersensitivity that meet our inclusion criteria. Of these 10 articles, eight articles report information about the association of HLA-B*1502 and phenytoin induced SJS/TEN. The results of these studies can be found in **Table 14**.

**Table 14:** HLA-B*1502 and phenytoin induced Stevens–Johnson syndrome (SJS)/toxic epidermal necrolysis (TEN).

Article	Population	Description	Sensitivity	Specificity	PPV	NPV	Incidence	NNG	Frequency carriers in population (%)
Chang et al. (2017)	Malaysia	16 SCAR cases (13 SJS/TEN, 3 DRESS) + 32 tolerant controls + 300 population controls	0.615	0.781	0.0067	0.9988	0.0024	677	15.7 (Chang et al., 2017)
Cheung et al. (2013)	Hong Kong Han Chinese	15 SJS/TEN cases + 75 controls	0.467	0.8	0.0056	0.9984	0.0024	893	17.9 (Hong Kong Chinese BMDR) (McCabe et al., 2020)
Hung et al. (2010)	Taiwan Han Chinese	26 SJS/TEN cases + 113 tolerant controls + 93 population controls	0.308	0.920	0.0092	0.9982	0.0024	1354	7.5 (Hung et al., 2010)
Locharernkul et al. (2008)	Thailand	16 SJS or MPE cases (4 SJS, 12 MPE) + 45 controls	1	0.822	0.0134	1	0.0024	417	16.1 (Thailand Northeast pop 2) (McCabe et al., 2020)
Shi et al. (2017)	China, Southern Han Chinese	13 SJS/TEN cases + 40 controls	0.462	0.775	0.0049	0.9983	0.0024	903	13.7 (China South Han) (McCabe et al., 2020)
Su et al. (2019)	Taiwan	128 SCAR (65 SJS/TEN, 63 DRESS) cases + 107 MPE cases + 376 controls	0.308	0.949	0.0144	0.9982	0.0024	1354	10.1 (Taiwan pop 2) (McCabe et al., 2020)
Tassaneeyakul et al. (2016)	Thailand	60 cases (39 SJS/TEN + 21 DRESS)+ 92 controls	0.128	0.859	0.0022	0.9976	0.0024	3250	16.1 (Thailand Northeast pop 2) (McCabe et al., 2020)
Yampayon et al. (2017)	Thailand	36 cases (15 SJS + 21 DRESS) + 100 tolerant controls + 758 population controls	0.333	0.820	0.0044	0.9980	0.0024	1250	14.2

The incidence of phenytoin induced SJS/TEN is assumed to be 0.24% in Asians and 0.069% in Caucasians (Fowler et al., 2019; Koninklijke Nederlandse Maatschappij ter bevordering der Pharmacie, 2020f).

The sensitivity of HLA-B*1502 for phenytoin induced SJS/TEN in the included studies differs from 12.8% to 100%. Of note, the study of Locharernkul et al. (2008) reporting a sensitivity of 100% has only four SJS cases. The second highest sensitivity found is only 61.5%. The specificity is 77.5%–94.9% which is quite low compared to the other drug-gene interactions in this review. The PPV is low (0.2%–1.4%) meaning false positives are common. The NNG to prevent one case of phenytoin induced SJS/TEN is 417–3,250.

## Discussion

In this review we provide a systematic overview of the diagnostic test criteria for HLA- genotyping to prevent drug hypersensitivity reactions. We have focused on the seven drugs for which actionable CPIC and/or DPWG guidelines are available. In general, specificity of all included HLA tests for drug hypersensitivity is high (80%–100%). Sensitivity shows a larger variability ranging from 100% for HLA-B*5701 testing and immunologically confirmed ABC-HSR to less than 30% for HLA-B*5701 testing and lamotrigine induced SJS/TEN. For allopurinol induced SCAR and HLA-B*5801 and flucloxacillin induced DILI and HLA-B*5701 sensitivity is high. For the other drugs and associated HLA variants, a wide range of sensitivities are found. Due to the rarity of some of the included hypersensitivity reactions, the NNG is very high for some drugs, especially for flucloxacillin and lamotrigine. Taking into consideration the low NNG of around 40 in combination with the severity of the side effect, it may not be surprising that HLA-B*1502 testing is mandatory for abacavir. Pre-emptively testing HLA-B*1502 for Asian carbamazepine initiators and HLA-B*5801 for high-risk allopurinol initiators could also be worthwhile.

To our knowledge, this is the first overview of the diagnostic test criteria of drug-HLA interactions for the actionable CPIC and/or DPWG guidelines. A review by Mullan et al. (2019) provides diagnostic test criteria but is limited to four AEDs. A strength of our study is that we calculated the diagnostic test criteria using the original data of genotyping results in cases and controls instead of only reporting diagnostic criteria if they were reported in the original studies. Also, some of the original studies did not take into account the incidence of the endpoint in the general population for calculating the NPV and PPV when this correction should be performed in case of a case-control design (Steinberg et al., 2009; Tonk et al., 2017).

A limitation of the available data is that most of the included studies have a limited sample size with mostly positive results, indicating a high likelihood of publication bias. Therefore presented results may be inflated and true effects may be lower than reported in this review. We included original studies which did not find a significant association of the variant but appeared significant in meta-analyses. This was particularly the case for lamotrigine where none of the selected original studies show a significant association of HLA-B*1502 and lamotrigine-induced SJS/TEN. However, in two meta-analyses, a significant association has been shown (Zeng et al., 2015; Deng et al., 2018). It must be noted however that also in these meta-analyses, the total number of patients was still low. The first meta-analysis had only 12 cases and 128 controls where the other meta-analysis had 54 cases and 313 controls. Besides the sample size of the studies, the number of studies found may also be a limitation. For flucloxacillin and oxcarbazepine results from only one study are available. Nevertheless, CPIC and/or DWPG released actionable guidelines for these gene-drug interactions and therefore the studies are included in this review. To better estimate the sensitivity, specificity, NPV, PPV and NNG, more and larger studies are warranted.

It should be noted that in the studies investigating HLA gene-drug interactions the studied endpoint is often a clinically diagnosed endpoint and the criteria of these endpoints may differ between studies. For example, the symptoms of ABC-HSR are nonspecific and may be difficult to objectify. In a study by Martin et al. (2004) data of some of the patients of the study by Mallal et al. (2002) are re-analyzed with stricter criteria for abacavir hypersensitivity. As a result Martin et al. reports a higher sensitivity of 94.7% instead of 77.8% in the original study. Also the difference of sensitivity between clinically diagnosed and immunologically diagnosed abacavir hypersensitivity further confirms the challenges related to the use of a clinical diagnosis as endpoint. Obviously, this has major influence on the estimated parameters such as sensitivity and specificity. When ABC-HSR is immunologically confirmed the sensitivity is 100%, however when clinically diagnosed, the sensitivity is much lower: 31%–90%. Also with conditions such as DRESS and SJS/TEN, the clinically diagnosed endpoint may differ between studies resulting in both over- or underestimation. The fact that clinically diagnosing hypersensitivity reactions is difficult is also exemplified by lamotrigine induced SJS. The drug label of lamotrigine states when 14 cases of serious rash associated with hospitalization were reviewed by three expert dermatologists, one dermatologist considered 7 of the 14 cases as SJS, while another dermatologist considered none of the cases to be SJS (GlaxoSmithKline, 2009). In many studies, SCAR is considered to be one endpoint even though it is a composite endpoint combining DRESS and SJS/TEN which are potentially related to two different biomarkers. Because of this, incidence rates of the separate conditions were sometimes hard to find. In this review we report numbers for allopurinol induced-SCAR but we also looked at DRESS and SJS/TEN separately. Sensitivity is high for both SJS/TEN and DRESS suggesting HLA-B*5801 is an appropriate biomarker for both endpoints.

For our analysis to calculate NPV, PPV and NNG for case-control studies, data on the incidence of the hypersensitivity reaction is needed. The incidence of drug hypersensitivity differs greatly between populations which can be explained by the differences in allele carrier frequencies. For example, the carrier frequency of HLA-B*5801 in the Portuguese population is approximately 4% while this is lower in most other European populations. In an Irish population and a German population for instance the carrier frequency of HLA-B*5801 is only 1%. Also in a Spanish population, geographically close to Portugal, the carrier frequency is already lower with 2.2%. This exemplifies that diagnostic test criteria should not be extrapolated. Since incidences of ADRs in specific populations are sometimes unknown, the best we could do is to assume these incidence figures from the general population. This assumption may influence the calculated NPV, PPV and NNG with the highest potential impact on the latter. The effect on the NPV and PPV will probably be low since NPV is already close to 1 and PPV close to 0. In these cases, we have made a best estimate based on results of incidence rates in similar populations. We used a systematic three-step approach. First, if available, we used the incidence mentioned in the original article. Next, we would take the incidence from the DPWG or CPIC guideline. Lastly, if no incidence or only a wide range was described in the guidelines, we used the incidence from a similar population derived from an original article. Therefore, we believe our study uses the most accurate incidence figures available for calculating NPV, PPV, and NNG.

Theoretically, in case of rare ADRs such as investigated here, population controls could be an alternative for the methodologically preferred tolerant controls. However, our search revealed the availability of tolerant controls for most gene-drug pairs and therefore we consistently chose using tolerant controls as to avoid confounding since mostly it is unknown if the ADR is also associated with disease susceptibility.

For a predictive HLA test to be implemented for pre-emptive testing, the NNG should be low, the clinical endpoint to be prevented should be of high severity and/or mortality and alternative treatments are available. The posterchild example of HLA testing is pre-emptive HLA-B*5701 testing for abacavir. The HLA-B*5701 test has a low NNG (~40). Besides, due to the high incidence of ABC-HSR, the test has a relatively high PPV of about 50%. Therefore when testing positive, there is a 50% chance the patient will develop abacavir hypersensitivity. The first symptoms of ABC-HSR are relatively mild composing of rash, fever, gastrointestinal disturbances and non-specific complaints as malaise, dizziness and headache. However, re-exposure is potentially fatal. Among patients who received abacavir in clinical trials, the mortality rate was 0.03 percent while the mortality among patients experiencing hypersensitivity symptoms after a re-challenge was around 5% (Hetherington et al., 2001). Also the mandatory testing as mentioned in the abacavir drug label has greatly stimulated HLA-B*5701 testing.

By pre-emptively testing patients who are at increased risk of developing hypersensitivity, a lower NNG can be reached. Therefore, based on our results we recommend to consider pre-emptively testing HLA-B*5801 in high risk allopurinol initiators and HLA-B*1502 in Asian carbamazepine initiators. The NNG for HLA-B*5801 and allopurinol induced SCAR is relatively low, approximately 500. However, due to the rarity of SCAR, the PPV is low. Therefore, allopurinol should only be withheld after a positive test result when alternative drugs for treatment are available. The NNG can be reduced 5–10 folds when testing only high risk patients such as patients with chronic renal insufficiency. Two studies find an incidence of allopurinol induced SCAR of 1%–2% in patients with chronic renal insufficiency, which is 5–10 times higher than the incidence in the overall allopurinol initiators (Jung et al., 2011; Park et al., 2019). By pre-emptively testing only patients with chronic renal insufficiency who are at higher risk of developing SCAR, the NNG would decrease to 50–100 patients.

For Asian carbamazepine initiators we consider the NNG of 500 to be low enough for pre-emptively testing. In this situation, the availability of alternative drugs for treatment is of major importance. When a patient is HLA-*1502 positive, the CPIC and/or DPWG recommendations advise to avoid the use of carbamazepine and to also avoid lamotrigine, phenytoin and oxcarbazepine when possible. Due to the rarity of the outcome, the positive predictive value is lower than 1% for HLA-B*1502 and SJS/TEN so false positives are common. This means for every 100 patients who switch to another AED, only one case of SJS/TEN is prevented while the alternative therapy may be suboptimal for the particular patient. The NNG can be lower than 500 when testing only in patients who are at higher risk such as patients with an Human immunodeficiency virus (HIV) infection. Patients with HIV infection have been reported to have a 100-fold higher risk of SJS/TEN than the general population. Also patients with active malignancy have an increased risk of SJS/TEN (High, 2020).

The aim of our review is to give an overview of diagnostic test criteria and this may help clinicians to decide which HLA tests could be implemented. The DPWG introduced the clinical implication score to assist this decision (Swen et al., 2018). The clinical implication score takes into account the clinical effect of the drug-gene interaction, the level of evidence, the NNG and pharmacogenetics information included in the drug label. Drug-gene pairs can be scored as essential, beneficial and potentially beneficial. When scored as essential, the DPWG concludes genotyping must be performed before initiation of drug therapy. For beneficial drug-gene interactions the DPWG recommends genotyping the patients before or directly after initiation of drug therapy. When scored as potentially beneficial, the DPWG states genotyping should be considered on an individual patient basis, but when genetic information is available, they recommend adhering to the guideline. The DPWG considers HLA-B*5701 testing essential for abacavir. HLA-B*1502 testing for oxcarbazepine and lamotrigine were scored to be beneficial. Other HLA-drug interactions have not yet been given a clinical implication score.

In this review we provide a systematic overview of the diagnostic test criteria for actionable drug-HLA gene interactions. In general, specificity and NPV of the HLA tests to predict drug hypersensitivity reactions are high whereas sensitivity shows a wide range across the different tests, ranging from 0-33% for HLA-B*1502 testing to predict lamotrigine induced SJS/TEN up to 100% for HLA-B*5701 to predict immunologically confirmed ABC-HSR. PPV is low for all tests where HLA-B*5701 testing for abacavir has the highest PPV of approximately 50%. The NNG is low for HLA-B testing for flucloxacillin and lamotrigine. HLA-B*5701 testing to predict ABC-HSR shows the lowest NNG followed by HLA-B*5801 for allopurinol induced SCAR and HLA-B*1502 for carbamazepine induced SJS/TEN.

## Author Contributions

LM performed the literature review and systemic analysis and contributed to writing the manuscript. JS and H-JG contributed to writing the manuscript. All authors contributed to the article and approved the submitted version.

## Funding

The research leading to these results has received funding from the European Community’s Horizon 2020 Programme under grant agreement No. 668353 (U-PGx).

## Conflict of Interest

The authors declare that the research was conducted in the absence of any commercial or financial relationships that could be construed as a potential conflict of interest.

The reviewer UA declared a past collaboration with one of the authors JS to the handling editor.
