# Supplementary material for: Diagnostic Test Criteria for HLA Genotyping to Prevent Drug Hypersensitivity Reactions: A Systematic Review of Actionable HLA Recommendations in CPIC and DPWG Guidelines
Source: Front Pharmacol. 2020 Sep 23;11:567048. doi: 10.3389/fphar.2020.567048 (PMC7538700; doi:10.3389/fphar.2020.567048)
Supplement: Supplementary file 1 [file DataSheet_1.docx]

# Supplementary file

Three consecutive searches have been performed, one for abacavir (as a test search), one for the four antiepileptic drugs and finally one for all other drugs associated with HLA-associated hypersensitivity reactions based on the HLA clinical decision support tool. [Bron] From these, we selected the drug-gene combinations designated as ‘actionable’ by the DPWG or CPIC.

All searches consisted of four different parts where only the first ‘drug related’ parts differed between the searches. The searched consisted of the following search terms including synonyms: ‘Drug’ AND ‘HLA’AND ‘hypersensitivity’ AND filters.

In total 1383 articles were retrieved in the three searches.

**Abacavir:**

**341 references on 20^th^ of November 2019**

**(("**abacavir"[Supplementary Concept] OR "abacavir"[tw] OR abacavir*[tw] OR "1592U89"[tw] OR "Ziagen"[tw] OR "(1S,4R)-4-(2-amino-6-(cyclopropylamino)-9H-purin-9-yl)-2-cyclopentene-1-methanol"[tw]) **AND** ("HLA Antigens"[Mesh] OR "HLA Antigens"[tw] OR "HLA Antigen"[tw] OR "HLA"[tw] OR "HLA-A"[tw] OR "HLA-A1"[tw] OR "HLA-A11"[tw] OR "HLA-A2"[tw] OR "HLA-A24"[tw] OR "HLA-A3"[tw] OR "HLA-B"[tw] OR "HLA-B13"[tw] OR "HLA-B14"[tw] OR "HLA-B15"[tw] OR "HLA-B18"[tw] OR "HLA-B27"[tw] OR "HLA-B35"[tw] OR "HLA-B37"[tw] OR "HLA-B38"[tw] OR "HLA-B39"[tw] OR "HLA-B40"[tw] OR "HLA-B44"[tw] OR "HLA-B51"[tw] OR "HLA-B52"[tw] OR "HLA-B7"[tw] OR "HLA-B8"[tw] OR "HLA-C"[tw] OR "HLA-D"[tw] OR "HLA-DP"[tw] OR "HLA-DQ"[tw] OR "HLA-DR"[tw] OR "HLA-DRB1"[tw] OR "HLA-DRB3"[tw] OR "HLA-DRB4"[tw] OR "HLA-DRB5"[tw] OR "HLA-DR1"[tw] OR "HLA-DR2"[tw] OR "HLA-DR3"[tw] OR "HLA-DR4"[tw] OR "HLA-DR5"[tw] OR "HLA-DR6"[tw] OR "HLA-DR7"[tw] OR "HLA-G"[tw] OR HLA-DQ*[tw] OR HLA-DR*[tw] OR "Human Leukocyte Antigens"[tw] OR "Human Leukocyte Antigen"[tw] OR "Human Leucocyte Antigens"[tw] OR "Human Leucocyte Antigen"[tw]) AND ("Drug Hypersensitivity"[mesh] OR "Drug Hypersensitivity"[tw] OR Drug Hypersensitiv*[tw] OR drug oversensitiv*[tw] OR drug allerg*[tw] OR drug eruption*[tw] OR "Aspirin-Induced Asthma"[tw] OR "Acute Generalized Exanthematous Pustulosis"[tw] OR "Erythema Nodosum"[tw] OR "Nicolau Syndrome"[tw] OR "Serum Sickness"[tw] OR "Stevens-Johnson Syndrome"[tw] OR hypersensitiv*[tw] OR oversensitiv*[tw] OR anaphyla*[tw] **OR "liver injury"[tw] OR hepatotox*[tw] OR "adverse effects"[subheading])** AND (english[la] OR dutch[la] OR chinese[la]) NOT ("Animals"[mesh] NOT "Humans"[mesh]) NOT **("veterinary"[ti] OR "rabbit"[ti] OR "rabbits"[ti] OR "animal"[ti] OR "animals"[ti] OR "mouse"[ti] OR "mice"[ti] OR "rodent"[ti] OR "rodents"[ti] OR "rat"[ti] OR "rats"[ti] OR "pig"[ti] OR "pigs"[ti] OR "porcine"[ti] OR "horse"[ti] OR "horses"[ti] OR "equine"[ti] OR "cow"[ti] OR "cows"[ti] OR "bovine"[ti] OR "goat"[ti] OR "goats"[ti] OR "sheep"[ti] OR "ovine"[ti] OR "canine"[ti] OR "dog"[ti] OR "dogs"[ti] OR "feline"[ti] OR "cat"[ti] OR "cats"[ti]) NOT** (("Review"[ptyp] OR "review"[ti] OR "Case Reports"[ptyp] OR "case report"[ti] OR "editorial"[ptyp] OR "editorial"[ti] OR "comment"[ptyp]) NOT ("Clinical Study"[ptyp] OR "trial"[ti] OR "RCT"[ti])))

**Carbamazepine, lamotrigine, oxcarbazepine and phenytoin:**

**372 references on 31^st^ of January 2020.**

(("Carbamazepine"[Mesh] OR "Oxcarbazepine"[Mesh] OR "Lamotrigine"[Mesh] OR "oxcarbamazepine"[Supplementary Concept] OR "Phenytoin"[Mesh] OR "carbamazepine"[tw] OR "Epitol"[tw] OR "Finlepsin"[tw] OR "Neurotol"[tw] OR "Tegretol"[tw] OR "Amizepine"[tw] OR "Oxcarbazepine"[tw] OR "10,11-Dihydro-10-oxo-5H-dibenz(b,f)azepine-5-carboxamide"[tw] OR "Trileptal"[tw] OR "Timox"[tw] OR "GP 47680"[tw] OR "Lamotrigine"[tw] OR "3,5-Diamino-6-(2,3-dichlorophenyl)-1,2,4-triazine"[tw] OR "Lamictal"[tw] OR "Lamiktal"[tw] OR "BW-430C"[tw] OR "BW 430C"[tw] OR "BW430C"[tw] OR "OX-CBZ"[tw] OR "phenytoin"[tw] OR "5,5-Diphenylhydantoin"[tw] OR "Fenitoin"[tw] OR "5,5-diphenylimidazolidine-2,4-dione"[tw] OR "Diphenylhydantoin"[tw] OR "Difenin"[tw] OR "Dihydan"[tw] OR "Phenytoin Sodium"[tw] OR "Sodium Diphenylhydantoinate"[tw] OR "Sodium Diphenylhydantoinate"[tw] OR "Epamin"[tw] OR "Epanutin"[tw] OR "Hydantol"[tw] OR "Antisacer"[tw] OR "Dilantin"[tw] OR carbamazepin*[tw] OR lamotrigin*[tw] OR oxcarbamazepin*[tw] OR phenytoin*[tw]) **AND** ("HLA Antigens"[Mesh] OR "HLA Antigens"[tw] OR "HLA Antigen"[tw] OR "HLA"[tw] OR "HLA-A"[tw] OR "HLA-A1"[tw] OR "HLA-A11"[tw] OR "HLA-A2"[tw] OR "HLA-A24"[tw] OR "HLA-A3"[tw] OR "HLA-B"[tw] OR "HLA-B13"[tw] OR "HLA-B14"[tw] OR "HLA-B15"[tw] OR "HLA-B18"[tw] OR "HLA-B27"[tw] OR "HLA-B35"[tw] OR "HLA-B37"[tw] OR "HLA-B38"[tw] OR "HLA-B39"[tw] OR "HLA-B40"[tw] OR "HLA-B44"[tw] OR "HLA-B51"[tw] OR "HLA-B52"[tw] OR "HLA-B7"[tw] OR "HLA-B8"[tw] OR "HLA-C"[tw] OR "HLA-D"[tw] OR "HLA-DP"[tw] OR "HLA-DQ"[tw] OR "HLA-DR"[tw] OR "HLA-DRB1"[tw] OR "HLA-DRB3"[tw] OR "HLA-DRB4"[tw] OR "HLA-DRB5"[tw] OR "HLA-DR1"[tw] OR "HLA-DR2"[tw] OR "HLA-DR3"[tw] OR "HLA-DR4"[tw] OR "HLA-DR5"[tw] OR "HLA-DR6"[tw] OR "HLA-DR7"[tw] OR "HLA-G"[tw] OR HLA-DQ*[tw] OR HLA-DR*[tw] OR "Human Leukocyte Antigens"[tw] OR "Human Leukocyte Antigen"[tw] OR "Human Leucocyte Antigens"[tw] OR "Human Leucocyte Antigen"[tw]) AND ("Drug Hypersensitivity"[mesh] OR "Drug Hypersensitivity"[tw] OR Drug Hypersensitiv*[tw] OR drug oversensitiv*[tw] OR drug allerg*[tw] OR drug eruption*[tw] OR "Aspirin-Induced Asthma"[tw] OR "Acute Generalized Exanthematous Pustulosis"[tw] OR "Erythema Nodosum"[tw] OR "Nicolau Syndrome"[tw] OR "Serum Sickness"[tw] OR "Stevens-Johnson Syndrome"[tw] OR hypersensitiv*[tw] OR oversensitiv*[tw] OR anaphyla*[tw] **OR "liver injury"[tw] OR hepatotox*[tw] OR "adverse effects"[subheading])** AND (english[la] OR dutch[la] OR chinese[la]) NOT ("Animals"[mesh] NOT "Humans"[mesh]) NOT **("veterinary"[ti] OR "rabbit"[ti] OR "rabbits"[ti] OR "animal"[ti] OR "animals"[ti] OR "mouse"[ti] OR "mice"[ti] OR "rodent"[ti] OR "rodents"[ti] OR "rat"[ti] OR "rats"[ti] OR "pig"[ti] OR "pigs"[ti] OR "porcine"[ti] OR "horse"[ti] OR "horses"[ti] OR "equine"[ti] OR "cow"[ti] OR "cows"[ti] OR "bovine"[ti] OR "goat"[ti] OR "goats"[ti] OR "sheep"[ti] OR "ovine"[ti] OR "canine"[ti] OR "dog"[ti] OR "dogs"[ti] OR "feline"[ti] OR "cat"[ti] OR "cats"[ti]) NOT** (("Review"[ptyp] OR "review"[ti] OR "Case Reports"[ptyp] OR "case report"[ti] OR "editorial"[ptyp] OR "editorial"[ti] OR "comment"[ptyp]) NOT ("Clinical Study"[ptyp] OR "trial"[ti] OR "RCT"[ti])))

**Other drugs associated with HLA associated hypersensitivity reactions (including allopurinol and flucloxacillin)**

**668 references on 21st of February 2020**

(("Allopurinol"[Mesh] OR "Amoxicillin-Potassium Clavulanate Combination"[Mesh] OR "Antitubercular Agents"[Mesh] OR "Antitubercular Agents"[Pharmacological Action] OR "Aspirin"[Mesh] OR "Clozapine"[Mesh] OR "Dapsone"[Mesh] OR "Floxacillin"[Mesh] OR "Lapatinib"[Mesh] OR "lumiracoxib"[Supplementary Concept] OR "Nevirapine"[Mesh] OR "Hydroxymethylglutaryl-CoA Reductase Inhibitors"[Mesh] OR "Hydroxymethylglutaryl-CoA Reductase Inhibitors"[Pharmacological Action] OR "Sulfamethoxazole"[Mesh] OR "Sulfasalazine"[Mesh] OR "Ticlopidine"[Mesh] OR "ximelagatran"[Supplementary Concept] OR "Vancomycin"[Mesh] OR "Infliximab"[Mesh] OR "allopurinol"[tw] OR "amoxicillin-clavulanate"[tw] OR "antituberculosis drugs"[tw] OR "aspirin"[tw] OR "clozapine"[tw] OR "dapsone"[tw] OR "flucloxacillin"[tw] OR "Floxacillin"[tw] OR "lapatinib"[tw] OR "lumiracoxib"[tw] OR "nevirapine"[tw] OR "statins"[tw] OR "statin"[tw] OR "Hydroxymethylglutaryl-CoA Reductase Inhibitor"[tw] OR "Hydroxymethylglutaryl-CoA Reductase Inhibitors"[tw] OR "sulfamethoxazole"[tw] OR "sulfasalazine"[tw] OR "ticlopidine"[tw] OR "ximelagatran"[tw] OR "vancomycin"[tw] OR "infliximab"[tw] OR "Anti-Inflammatory Agents, Non-Steroidal"[Mesh] OR "Anti-Inflammatory Agents, Non-Steroidal"[Pharmacological Action] OR "Non-Steroidal Anti-Inflammatory Agents"[tw] OR "Non-Steroidal Anti-Inflammatory Agents"[tw] OR "NSAID"[tw] OR "NSAIDs"[tw] OR "Uribenz"[tw] OR "Allural"[tw] OR "Apulonga"[tw] OR "Apurin"[tw] OR "Atisuril"[tw] OR "Bleminol"[tw] OR "Caplenal"[tw] OR "Cellidrin"[tw] OR "Embarin"[tw] OR "Suspendol"[tw] OR "Foligan"[tw] OR "Hamarin"[tw] OR "Lopurin"[tw] OR "Lysuron"[tw] OR "Milurit"[tw] OR "Milurite"[tw] OR "Uripurinol"[tw] OR "Urosin"[tw] OR "Urtias"[tw] OR "Zyloprim"[tw] OR "Zyloric"[tw] OR "Purinol"[tw] OR "Progout"[tw] OR "Remid"[tw] OR "Amoxicillin Potassium Clavulanate Combination"[tw] OR "Co-amoxiclav"[tw] OR "Co amoxiclav"[tw] OR "Coamoxiclav"[tw] OR "Amoxicillin-Clavulanic Acid"[tw] OR "Amoxicillin Clavulanic Acid"[tw] OR "Amoxi-Clavulanate"[tw] OR "Amoxi Clavulanate"[tw] OR "Amoxycillin-Clavulanic Acid"[tw] OR "Amoxycillin Clavulanic Acid"[tw] OR "Amox-clav"[tw] OR "Amox clav"[tw] OR "Clavulanate Potentiated Amoxycillin"[tw] OR "BRL-25000"[tw] OR "BRL 25000"[tw] OR "BRL25000"[tw] OR "Synulox"[tw] OR "Spektramox"[tw] OR "Augmentin"[tw] OR "Clavulin"[tw] OR "aconiazide"[tw] OR "Aminosalicylic Acid"[tw] OR "amprenavir"[tw] OR "bedaquiline"[tw] OR "Capreomycin"[tw] OR "Cycloserine"[tw] OR "Diarylquinolines"[tw] OR "Enviomycin"[tw] OR "essential 303 forte"[tw] OR "Ethambutol"[tw] OR "Ethionamide"[tw] OR "Isoniazid"[tw] OR "isoniazid, pyrazinamide, rifampin drug combination"[tw] OR "KRM 1648"[tw] OR "macozinone"[tw] OR "Mycophenolic Acid"[tw] OR "pazufloxacin"[tw] OR "Prothionamide"[tw] OR "Pyrazinamide"[tw] OR "Rifabutin"[tw] OR "Rifampin"[tw] OR "rifapentine"[tw] OR "sodium thiosulfate"[tw] OR "sparfloxacin"[tw] OR "Thioacetazone"[tw] OR "thiobenzamide"[tw] OR "thiocarlide"[tw] OR "thymopoietin III"[tw] OR "Viomycin"[tw] OR "Acetylsalicylic Acid"[tw] OR "Acid, Acetylsalicylic"[tw] OR "2-(Acetyloxy)benzoic Acid"[tw] OR "Acylpyrin"[tw] OR "Colfarit"[tw] OR "Easprin"[tw] OR "Ecotrin"[tw] OR "Endosprin"[tw] OR "Magnecyl"[tw] OR "Micristin"[tw] OR "Polopirin"[tw] OR "Polopiryna"[tw] OR "Solprin"[tw] OR "Solupsan"[tw] OR "Zorprin"[tw] OR "Clozaril"[tw] OR "Leponex"[tw] OR "DADPS"[tw] OR "Sulfonyldianiline"[tw] OR "Diaminodiphenylsulfone"[tw] OR "Diaphenylsulfone"[tw] OR "Sulfona"[tw] OR "Dapson-Fatol"[tw] OR "Disulone"[tw] OR "Avlosulfone"[tw] OR "Tykerb"[tw] OR "GW282974X"[tw] OR "GW572016"[tw] OR "GW-572016"[tw] OR "GW 572016"[tw] OR "prexige"[tw] OR "COX 189"[tw] OR "COX-189"[tw] OR "Viramune"[tw] OR "BI-RG-587"[tw] OR "BI RG 587"[tw] OR "BIRG587"[tw] OR "2,6,7-trideoxy-7-C-(2,4-dichlorophenyl)heptonic acid"[tw] OR "3,24-dihydroxycholest-8(14)-en-15-one"[tw] OR "3,25-dihydroxycholest-8(14)-en-15-one"[tw] OR "3,4-dihydroxyphenylpropionylleucinamide"[tw] OR "3-hydroxy-24-dimethylaminochol-8(14)-en-15-one"[tw] OR "3-hydroxy-25,26,26,26,27,27,27-heptafluoro-5-cholest-8(14)-en-15-one"[tw] OR "3-hydroxycholest-8(14),24-dien-15-one"[tw] OR "6-(2,4-dichlorophenyl)-2,3,4-trihydroxyhexanesulfonic acid"[tw] OR "6-(2,4-dichlorophenyl)-2,4-dihydroxyhexane-1-sulfonic acid"[tw] OR "6-(2,4-dichlorophenyl)-erythro-2,4-dihydroxyhexylphosphonic acid"[tw] OR "6-hydroxyisocompactin"[tw] OR "7-(2,4-dichlorophenyl)-3-hydroxy-5-heptanolide"[tw] OR "amlodipine, atorvastatin drug combination"[tw] OR "Atorvastatin"[tw] OR "BMY 21950"[tw] OR "cerivastatin"[tw] OR "cholest-8-ene-3,15-diol"[tw] OR "chrysosporin"[tw] OR "crilvastatin"[tw] OR "dihydrocompactin"[tw] OR "dihydromevinolin"[tw] OR "fermodulin"[tw] OR "GR 92549"[tw] OR "GR 95030X"[tw] OR "HR 780"[tw] OR "L 157012"[tw] OR "L 645164"[tw] OR "L 647318"[tw] OR "L 669262"[tw] OR "lanost-8-en-3-ol-24-one"[tw] OR "Lovastatin"[tw] OR "Meglutol"[tw] OR "methoxypolyethoxylated cholesterol"[tw] OR "mevastatin"[tw] OR "pannorin"[tw] OR "PD 123244-15"[tw] OR "phosphoadenosine diphosphoribose"[tw] OR "pitavastatin"[tw] OR "Pravastatin"[tw] OR "red yeast rice"[tw] OR "Rosuvastatin Calcium"[tw] OR "RP 61969"[tw] OR "Simvastatin"[tw] OR "SQ 33600"[tw] OR "SRI 62320"[tw] OR "Sulphamethoxazole"[tw] OR "Sulfamethylisoxazole"[tw] OR "Sulfisomezole"[tw] OR "Gantanol"[tw] OR "Salicylazosulfapyridine"[tw] OR "Sulphasalazine"[tw] OR "Salazosulfapyridine"[tw] OR "Azulfidine EN"[tw] OR "Azulfidine"[tw] OR "Asulfidine"[tw] OR "Colo-Pleon"[tw] OR "Colo Pleon"[tw] OR "Pleon"[tw] OR "Ucine"[tw] OR "Salazopyrin"[tw] OR "Ticlodone"[tw] OR "5332C"[tw] OR "Ticlid"[tw] OR "xi-melagatran"[tw] OR "Exanta"[tw] OR "H 376 95"[tw] OR "H 376-95"[tw] OR "Vancocin"[tw] OR "Vancocine"[tw] OR "Vancomicina"[tw] OR "Monoclonal Antibody cA2"[tw] OR "MAb cA2"[tw] OR "Infliximab-abda"[tw] OR "Renflexis"[tw] OR "Infliximab-dyyb"[tw] OR "Inflectra"[tw] OR "Remicade"[tw] OR "1-((4,5-bis(4-methoxyphenyl)-2-thiazoyl)carbonyl)-4-methylpiperazine"[tw] OR "1-((4-methylsulfonyl)phenyl)-3-trifluoromethyl-5-(4-fluorophenyl)pyrazole"[tw] OR "1-(4-chlorobenzoyl)-3-(2-(1H-imidazol-1-yl)-2-oxoethyl)-5-methoxy-2-methyl-1H-indole"[tw] OR "2-(4-(quinolin-2-yl-methoxy)phenyl)-2-cyclopentylacetic acid"[tw] OR "2-(4-acetoxyphenyl)-2-chloro-N-methylethylamine"[tw] OR "2-aminomethyl-4-t-butyl-6-iodophenol"[tw] OR "2-diethylaminoethanol"[tw] OR "4,5-Dihydro-1-(3-(trifluoromethyl)phenyl)-1H-pyrazol-3-amine"[tw] OR "4-(5-(4-chlorophenyl)-3-(trifluoromethyl)-1H-pyrazol-1-yl)benzenesulfonamide"[tw] OR "4-bromo-2,7-dimethoxy-3H-phenothiazin-3-one"[tw] OR "6-(4-fluorophenyl)-2,3-dihydro-5-(4-pyridinyl)imidazo(2,1-b)thiazole"[tw] OR "6-acetylaminocaproic acid"[tw] OR "6-ethoxy-3-(4-methanesulfonylphenyl)-4-phenylpyran-2-one"[tw] OR "7-methoxy-alpha-methyl-2-naphthaleneacetic acid"[tw] OR "A 771726"[tw] OR "aceclofenac"[tw] OR "acemetacin"[tw] OR "acetaminophen, aspirin, caffeine drug combination"[tw] OR "acetaminophen, butalbital, caffeine drug combination"[tw] OR "acetaminophen, hydrocodone drug combination"[tw] OR "acetosyringone"[tw] OR "acetovanillone"[tw] OR "acetylsalicylic acid lysinate"[tw] OR "Adapalene"[tw] OR "Adapalene, Benzoyl Peroxide Drug Combination"[tw] OR "alclofenac"[tw] OR "alminoprofen"[tw] OR "alpha-pentyl-3-(2-quinolinylmethoxy)benzenemethanol"[tw] OR "amiprilose"[tw] OR "Ampyrone"[tw] OR "amylase, phosphates, proteases drug combinations"[tw] OR "andrographolide"[tw] OR "anisodamine"[tw] OR "anisodine"[tw] OR "antiflammin P2"[tw] OR "Antipyrine"[tw] OR "Apazone"[tw] OR "apremilast"[tw] OR "Arteparon"[tw] OR "Arthrotec"[tw] OR "Aspirin"[tw] OR "aspirin, aluminum hydroxide, magnesium hydroxide drug combination"[tw] OR "aspirin, butalbital and caffeine drug combination"[tw] OR "aspirin, meprobamate drug combination"[tw] OR "atrinositol"[tw] OR "azulene"[tw] OR "baicalin"[tw] OR "balsalazide"[tw] OR "bendazac"[tw] OR "bendazac lysine"[tw] OR "benorilate"[tw] OR "benoxaprofen"[tw] OR "benzobarbital"[tw] OR "berbamine"[tw] OR "betulinic acid"[tw] OR "bevonium"[tw] OR "BI 607812 BS"[tw] OR "biphenylylacetic acid"[tw] OR "boldine"[tw] OR "boswellic acid"[tw] OR "bromfenac"[tw] OR "bucillamine"[tw] OR "Bufexamac"[tw] OR "bumadizone"[tw] OR "butibufen"[tw] OR "carbaspirin calcium"[tw] OR "carprofen"[tw] OR "caryophyllene"[tw] OR "castanospermine"[tw] OR "CDP 571"[tw] OR "Celecoxib"[tw] OR "cepharanthine"[tw] OR "chloroquine diphosphate"[tw] OR "choline magnesium trisalicylate"[tw] OR "chrysarobin"[tw] OR "Clonixin"[tw] OR "CP 96345"[tw] OR "Curcumin"[tw] OR "CX 659S"[tw] OR "dauricine"[tw] OR "dexketoprofen trometamol"[tw] OR "Diclofenac"[tw] OR "diclofenac hydroxyethylpyrrolidine"[tw] OR "difenpiramide"[tw] OR "Diflunisal"[tw] OR "dimephosphon"[tw] OR "Dipyrone"[tw] OR "diucifon"[tw] OR "droxicam"[tw] OR "DuP 697"[tw] OR "ebselen"[tw] OR "ecallantide"[tw] OR "eltenac"[tw] OR "enfenamic acid"[tw] OR "enkephalin-Leu, Ala(2)-Arg(6)-"[tw] OR "Epirizole"[tw] OR "Etanercept"[tw] OR "ethenzamide"[tw] OR "Ethonium"[tw] OR "Etodolac"[tw] OR "etofenamate"[tw] OR "Etoricoxib"[tw] OR "evening primrose oil"[tw] OR "fenamic acid"[tw] OR "fenbufen"[tw] OR "fenclofenac"[tw] OR "fenflumizole"[tw] OR "Fenoprofen"[tw] OR "fentiazac"[tw] OR "fepradinol"[tw] OR "Feprazone"[tw] OR "ferulic acid"[tw] OR "floctafenine"[tw] OR "flosulide"[tw] OR "flunixin"[tw] OR "flunixin meglumine"[tw] OR "flunoxaprofen"[tw] OR "fluproquazone"[tw] OR "Flurbiprofen"[tw] OR "flurbiprofen axetil"[tw] OR "FR 167653"[tw] OR "FR 173657"[tw] OR "glucametacin"[tw] OR "guacetisal"[tw] OR "helenalin"[tw] OR "heliodermin"[tw] OR "hemodes"[tw] OR "higenamine"[tw] OR "Ibuprofen"[tw] OR "ibuproxam"[tw] OR "icatibant"[tw] OR "IH 764-3"[tw] OR "imidazole-2-hydroxybenzoate"[tw] OR "indobufen"[tw] OR "Indomethacin"[tw] OR "Indoprofen"[tw] OR "iodoantipyrine"[tw] OR "isoxicam"[tw] OR "kebuzone"[tw] OR "Ketoprofen"[tw] OR "ketoprofen lysine"[tw] OR "Ketorolac"[tw] OR "Ketorolac Tromethamine"[tw] OR "L 745337"[tw] OR "L 778736"[tw] OR "licofelone"[tw] OR "lipoxin A4"[tw] OR "lipoxin B4"[tw] OR "lisofylline"[tw] OR "lobenzarit"[tw] OR "lonazolac"[tw] OR "lornoxicam"[tw] OR "loxoprofen"[tw] OR "LQFM-091"[tw] OR "lumiracoxib"[tw] OR "Magnesium Salicylate"[tw] OR "magnolol"[tw] OR "manoalide"[tw] OR "Masoprocol"[tw] OR "Meclofenamic Acid"[tw] OR "Mefenamic Acid"[tw] OR "Meloxicam"[tw] OR "Mesalamine"[tw] OR "mizoribine"[tw] OR "mofebutazone"[tw] OR "mofezolac"[tw] OR "N-(2-cyclohexyloxy-4-nitrophenyl)methanesulfonamide"[tw] OR "N-(9H-(2,7-dimethylfluoren-9-ylmethoxy)carbonyl)leucine"[tw] OR "N-succinimidyl-1-(4-chlorobenzoyl)-5-methoxy-2-methyl-1H-indole-3-acetate"[tw] OR "Nabumetone"[tw] OR "nafamostat"[tw] OR "Naproxen"[tw] OR "Nebacetin"[tw] OR "nepafenac"[tw] OR "nifenazone"[tw] OR "Niflumic Acid"[tw] OR "nimesulide"[tw] OR "nitroaspirin"[tw] OR "Olopatadine Hydrochloride"[tw] OR "olsalazine"[tw] OR "olvanil"[tw] OR "oren gedoku to"[tw] OR "orgotein"[tw] OR "Oxaprozin"[tw] OR "Oxyphenbutazone"[tw] OR "palmidrol"[tw] OR "parecoxib"[tw] OR "parthenolide"[tw] OR "peoniflorin"[tw] OR "phenidone"[tw] OR "Phenylbutazone"[tw] OR "pimecrolimus"[tw] OR "pirfenidone"[tw] OR "Piroxicam"[tw] OR "piroxicam-beta-cyclodextrin"[tw] OR "pirprofen"[tw] OR "proglumetacin"[tw] OR "propacetamol"[tw] OR "propionylcarnitine"[tw] OR "propyphenazone"[tw] OR "proquazone"[tw] OR "pyranoprofen"[tw] OR "pyrazolone"[tw] OR "pyrogenal"[tw] OR "RNS60"[tw] OR "rofecoxib"[tw] OR "rosmarinic acid"[tw] OR "Rumalon"[tw] OR "saiko-keishi-to"[tw] OR "saikosaponin"[tw] OR "salicin"[tw] OR "salicylamide"[tw] OR "Salicylates"[tw] OR "salicylsalicylic acid"[tw] OR "SB 203580"[tw] OR "SC 299"[tw] OR "SC 41930"[tw] OR "SC 560"[tw] OR "semapimod"[tw] OR "seratrodast"[tw] OR "serratiopeptidase"[tw] OR "shikonin"[tw] OR "sinapaldehyde"[tw] OR "Sodium Salicylate"[tw] OR "ST 679"[tw] OR "Sul-121"[tw] OR "Sulfasalazine"[tw] OR "Sulindac"[tw] OR "sulindac sulfide"[tw] OR "sulindac sulfone"[tw] OR "Suprofen"[tw] OR "suxibuzone"[tw] OR "tanshinone"[tw] OR "taxifolin"[tw] OR "tenidap"[tw] OR "tenoxicam"[tw] OR "tepoxalin"[tw] OR "tiaprofenic acid"[tw] OR "tiaramide"[tw] OR "tinoridine"[tw] OR "tolfenamic acid"[tw] OR "Tolmetin"[tw] OR "tramadol, dexketoprofen drug combination"[tw] OR "tranilast"[tw] OR "tribenoside"[tw] OR "ursolic acid"[tw] OR "valdecoxib"[tw] OR "zileuton"[tw] OR "zomepirac"[tw] OR multi-ingredient cold*[tw]) **AND** ("HLA Antigens"[Mesh] OR "HLA Antigens"[tw] OR "HLA Antigen"[tw] OR "HLA"[tw] OR "HLA-A"[tw] OR "HLA-A1"[tw] OR "HLA-A11"[tw] OR "HLA-A2"[tw] OR "HLA-A24"[tw] OR "HLA-A3"[tw] OR "HLA-B"[tw] OR "HLA-B13"[tw] OR "HLA-B14"[tw] OR "HLA-B15"[tw] OR "HLA-B18"[tw] OR "HLA-B27"[tw] OR "HLA-B35"[tw] OR "HLA-B37"[tw] OR "HLA-B38"[tw] OR "HLA-B39"[tw] OR "HLA-B40"[tw] OR "HLA-B44"[tw] OR "HLA-B51"[tw] OR "HLA-B52"[tw] OR "HLA-B7"[tw] OR "HLA-B8"[tw] OR "HLA-C"[tw] OR "HLA-D"[tw] OR "HLA-DP"[tw] OR "HLA-DQ"[tw] OR "HLA-DR"[tw] OR "HLA-DRB1"[tw] OR "HLA-DRB3"[tw] OR "HLA-DRB4"[tw] OR "HLA-DRB5"[tw] OR "HLA-DR1"[tw] OR "HLA-DR2"[tw] OR "HLA-DR3"[tw] OR "HLA-DR4"[tw] OR "HLA-DR5"[tw] OR "HLA-DR6"[tw] OR "HLA-DR7"[tw] OR "HLA-G"[tw] OR HLA-DQ*[tw] OR HLA-DR*[tw] OR "Human Leukocyte Antigens"[tw] OR "Human Leukocyte Antigen"[tw] OR "Human Leucocyte Antigens"[tw] OR "Human Leucocyte Antigen"[tw]) AND ("Drug Hypersensitivity"[mesh] OR "Drug Hypersensitivity"[tw] OR Drug Hypersensitiv*[tw] OR drug oversensitiv*[tw] OR drug allerg*[tw] OR drug eruption*[tw] OR "Aspirin-Induced Asthma"[tw] OR "Acute Generalized Exanthematous Pustulosis"[tw] OR "Erythema Nodosum"[tw] OR "Nicolau Syndrome"[tw] OR "Serum Sickness"[tw] OR "Stevens-Johnson Syndrome"[tw] OR hypersensitiv*[tw] OR oversensitiv*[tw] OR anaphyla*[tw] **OR "liver injury"[tw] OR hepatotox*[tw] OR "adverse effects"[subheading])** AND (english[la] OR dutch[la] OR chinese[la]) NOT ("Animals"[mesh] NOT "Humans"[mesh]) NOT **("veterinary"[ti] OR "rabbit"[ti] OR "rabbits"[ti] OR "animal"[ti] OR "animals"[ti] OR "mouse"[ti] OR "mice"[ti] OR "rodent"[ti] OR "rodents"[ti] OR "rat"[ti] OR "rats"[ti] OR "pig"[ti] OR "pigs"[ti] OR "porcine"[ti] OR "horse"[ti] OR "horses"[ti] OR "equine"[ti] OR "cow"[ti] OR "cows"[ti] OR "bovine"[ti] OR "goat"[ti] OR "goats"[ti] OR "sheep"[ti] OR "ovine"[ti] OR "canine"[ti] OR "dog"[ti] OR "dogs"[ti] OR "feline"[ti] OR "cat"[ti] OR "cats"[ti]) NOT** (("Review"[ptyp] OR "review"[ti] OR "Case Reports"[ptyp] OR "case report"[ti] OR "editorial"[ptyp] OR "editorial"[ti] OR "comment"[ptyp]) NOT ("Clinical Study"[ptyp] OR "trial"[ti] OR "RCT"[ti])))
